# Proactive community case-finding to facilitate treatment seeking for mental disorders, Nepal

**DOI:** 10.2471/BLT.16.189282

**Published:** 2017-04-25

**Authors:** Mark JD Jordans, Brandon A Kohrt, Nagendra P Luitel, Crick Lund, Ivan H Komproe

**Affiliations:** a Centre for Global Mental Health, Institute of Psychiatry, Psychology and Neuroscience, King’s College London, 16 De Crespigny Park, Camberwell, London SE5 8AF, England.; b Duke Global Health Institute, Duke University, Durham, United States of America.; c Transcultural Psychosocial Organization, Kathmandu, Nepal.; d Alan J Fisher Centre for Public Mental Health, University of Cape Town, Cape Town, South Africa.; e Faculty of Social & Behavioural Sciences, Utrecht University, Utrecht, Netherlands.

## Abstract

**Problem:**

Underutilization of mental health services is a major barrier to reducing the burden of disease attributable to mental, neurological and substance-use disorders. Primary care-based screening to detect people with mental disorders misses people not frequently visiting health-care facilities or who lack access to services.

**Approach:**

In two districts in Nepal, we trained lay community informants to use a tool to detect people with mental, neurological and substance-use disorders during routine community service. The community informant detection tool consists of vignettes, which are sensitive to the context, and pictures that are easy to understand for low literacy populations. Informants referred people they identified using the tool to health-care facilities. Three weeks after detection, people were interviewed by trained research assistants to assess their help-seeking behaviour and whether they received any treatment.

**Local setting:**

Decentralized mental health services are scarce in Nepal and few people with mental disorders are seeking care.

**Relevant changes:**

Out of the 509 people identified through the community informant detection tool, two-thirds (67%; 341) accessed health services and 77% (264) of those individuals initiated mental health treatment. People in the rural Pyuthan district (208 out of 268) were more likely to access health care than those living in Chitwan district (133 out of 241).

**Lessons learnt:**

The introduction of the tool increased the utilization of mental health services in a low-income country with few health resources. The tool seems beneficial in rural settings, where communities are close-knit and community informants are familiar with those in need of mental health services.

## Résumé

**Problème:**

La sous-utilisation des services de santé mentale est un obstacle majeur à la réduction de la charge de morbidité attribuable aux troubles mentaux, neurologiques et associés à la toxicomanie. Le dépistage des troubles mentaux lors des soins primaires ne permet pas de les détecter chez les personnes qui viennent rarement en consultation dans des établissements de soins ou qui n'ont pas accès à ces services.

**Approche:**

Dans deux districts du Népal, nous avons formé des informateurs de la communauté non professionnels à l'utilisation d'un outil permettant de détecter les personnes atteintes de troubles mentaux, neurologiques et associés à la toxicomanie lors de services ordinaires dans la communauté. Cet outil comprenait des scénarios adaptés à différents contextes et des images facilement compréhensibles par des populations peu alphabétisées. Les informateurs ont orienté les personnes repérées à l'aide de cet outil vers des établissements de santé. Trois semaines plus tard, ces personnes ont été interrogées par des assistants de recherche formés afin d'évaluer leur demande de soins et de savoir si elles avaient reçu un traitement.

**Environnement local:**

Il existe peu de services de santé mentale décentralisés au Népal et peu de personnes atteintes de troubles mentaux ont recours à des soins.

**Changements significatifs:**

Sur les 509 personnes repérées grâce à l'outil de dépistage par des informateurs de la communauté, deux tiers (67%; 341) ont accédé à des services de santé et 77% (264) ont commencé un traitement pour leurs troubles mentaux. Les habitants du district rural de Pyuthan (208 sur 268) étaient plus nombreux à accéder aux soins que ceux du district de Chitwan (133 sur 241).

**Leçons tirées:**

L'introduction de l'outil a permis d'augmenter le recours aux services de santé mentale dans un pays à faible revenu disposant de peu de ressources en matière de santé. Cet outil semble bénéfique en milieu rural, où les communautés sont très unies et où les informateurs connaissent les personnes qui ont besoin de services de santé mentale.

## Resumen

**Situación:**

La infrautilización de servicios de salud mental es un gran impedimento para reducir la carga de enfermedades atribuibles a trastornos mentales, neurológicos y por consumo de sustancias. Los exámenes basados en atención primaria para detectar personas con trastornos mentales no incluyen a las personas que no visitan centros de salud frecuentemente o que no tienen acceso a los servicios.

**Enfoque:**

En dos distritos de Nepal, se formó a informantes comunitarios no profesionales para el uso de una herramienta que detecta a personas con trastornos mentales, neurológicos y del uso de sustancias durante un servicio comunitario rutinario. La herramienta de detección de los informantes comunitarios consiste de viñetas, que dependen del contexto, e imágenes fáciles de entender para poblaciones con un nivel de alfabetización bajo. Los informantes enviaron a las personas identificadas con la herramienta a centros sanitarios. Tres semanas después de la detección, asistentes de investigación con formación entrevistaron a estas personas para evaluar su comportamiento de búsqueda de ayuda y si recibieron algún tratamiento.

**Marco regional:**

Los servicios de salud mental descentralizados son muy escasos en Nepal y pocas personas con trastornos mentales buscan atención.

**Cambios importantes:**

De las 509 personas identificadas con la herramienta de detección de los informantes comunitarios, dos tercios (67%; 341) accedieron a servicios sanitarios y el 77% (264) de dichos individuos inició un tratamiento de salud mental. Las personas del distrito rural de Pyuthan (208 de 268) tuvieron más posibilidades de acceder a atención sanitaria que los que vivían en el distrito de Chitwan (133 de 241).

**Lecciones aprendidas:**

La introducción de la herramienta aumentó el uso de servicios de salud mental en un país con ingresos bajos y escasos recursos sanitarios. La herramienta parece ser beneficiosa en zonas rurales, donde las comunidades están muy cohesionadas y los informantes comunitarios están familiarizados con las personas que necesitan servicios de salud mental.

## ملخص

**المشكلة:**

يشكل عدم استخدام خدمات الصحة العقلية عائقًا رئيسيًا أمام الحد من عبء المرض الذي يعزى إلى الاضطرابات العقلية والعصبية والاضطرابات الناجمة عن تعاطي المخدرات. ويعيب الفحص الأولي القائم على الرعاية للكشف عن المصابين باضطرابات عقلية عدم قدرته على تمييز لأشخاص الذين لا يترددون على أماكن الرعاية الصحية الأولية بشكل متكرر أو الذين لا تتوفر لهم سبل الحصول على تلك الخدمات.

**الأسلوب:**

قمنا بتدريب كشافين محليين من أبناء المجتمع لاستخدام أداة لاكتشاف المصابين باضطرابات عقلية وعصبية واضطرابات تتعلق بإساءة استخدام المواد خلال الخدمة المجتمعية الروتينية في منطقتين في نيبال. تتكون أداة الكشف المخصصة للكشافين المجتمعيين من أشكال وصفية تخص السياق، وصور يسهل فهمها للقطاعات السكانية التي تسودها مستويات متدنية من التعليم. وقام الكشافون بإحالة الأشخاص الذين حددوهم باستخدام الأداة إلى مرافق الرعاية الصحية. وبعد ثلاثة أسابيع من الكشف، أجرى باحثون مُدرّبون مقابلات مع هؤلاء الأشخاص لتقييم سلوكهم في طلب المساعدة والتحقق مما إذا كانوا قد تلقوا أي علاج.

**المواقع المحلية:**

يندر وجود خدمات لامركزية للصحة العقلية في نيبال، كما يسعى عدد قليل من الأشخاص الذين يعانون من اضطرابات عقلية إلى الحصول على الرعاية.

**التغييرات ذات الصلة:**

من أصل 509 أشخاص تم التعرف عليهم من خلال أداة الكشف عبر جهود الكشافين، كان ثلثاهم (بواقع 67‏٪ أي 341 منهم) يتلقون الخدمات الصحية، فيما بدأ ‏77‏٪ (264) من هؤلاء الأفراد تلقي العلاج العقلي. وكان سكان منطقة بيوثان الريفية (208 من أصل 268) أكثر من حصل على الرعاية الصحية من أولئك الذين يعيشون في منطقة تشيتوان (133 من أصل 241).

**الدروس المستفادة:**

أدى تقديم هذه الأداة إلى زيادة الاستفادة من خدمات الصحة العقلية في بلد منخفض الدخل ويسوده الشح في الموارد الصحية. وتبدو هذه الأداة مفيدة في البيئات الريفية، حيث تكون المجتمعات المحلية متماسكة، ويكون الكشافون المجتمعيون على دراية بالمحتاجين إلى خدمات الصحة العقلية.

## 摘要

**问题:**

对精神卫生服务利用不足是减少精神、神经和物质使用障碍引起的疾病负担的主要障碍。 以初级医疗为基础的筛查在检测精神障碍患者时会遗漏不经常前往医疗保健机构就诊的人员或无法获得服务的人员。

**方法:**

在尼泊尔的两个地区，我们培训了没有经验的社区信息提供人员，以便在日常社区服务中使用工具来检测精神、神经和物质使用障碍患者。 社区信息检测工具包括对背景敏感的片段，以及对于识字率低的人群来说容易理解的图片。 信息提供人员将其使用工具确定的患者转介到医护机构。 检测后三周内，受过培训的研究助理人员对患者进行了访问，以评估他们的求助行为以及他们是否接受了任何治疗。

**当地状况:**

尼泊尔分散化的精神卫生服务很稀少并且很少有精神障碍患者求医。

**相关变化:**

在通过社区信息检测工具确定的 509 人中，有三分之二的人 (67%; 341) 获得了卫生服务并且其中 77% (264) 的人开始接受精神卫生治疗。 与居住在 Chitwan 地区的人员 (133/241) 相比，Pyuthan 农村地区的人员 (208/268) 更有可能获得医疗护理。

**经验教训:**

在医疗资源缺乏的低收入国家引入该工具提高了精神卫生服务的利用率。 在社区间联系紧密且社区信息提供人员熟悉需要精神卫生服务的人员的农村地区，该工具可发挥有利作用。

## Резюме

**Проблема:**

Недостаточно широкое использование служб охраны психического здоровья является одним из основных препятствий для снижения уровня заболеваемости психическими, неврологическими расстройствами, а также расстройствами, связанными с употреблением психоактивных веществ. Первичное скрининговое обследование с целью выявления людей с психическими расстройствами не охватывает людей, которые нечасто посещают медицинские учреждения или которые не имеют доступа к медико-санитарной помощи.

**Подход:**

В двух районах Непала мы обучили общественных информантов использованию инструмента для выявления людей с психическими, неврологическими расстройствами, а также расстройствами, связанными с употреблением психоактивных веществ во время обычных общественных работ. Инструмент выявления, который использовали общественные информанты, состоял из иллюстраций, соответствующих местным условиям и контексту, и изображений, которые являются понятными для групп с низкой грамотностью. Информанты направляли людей, выявленных с помощью этого инструмента, в медицинские учреждения. Через три недели квалифицированными ассистентами исследователей был проведен опрос выявленных людей с целью оценки их действий относительно получения медицинской помощи и выяснения того, получили ли они какое-либо лечение.

**Местные условия:**

В Непале не хватает децентрализованных служб охраны психического здоровья и лишь немногие люди с психическими расстройствами обращаются за помощью.

**Осуществленные перемены:**

Из 509 человек, выявленных с помощью инструмента общественных информантов, две трети (67%; 341) обратились в медицинские службы, 77% (264) из этих лиц начали лечение психического расстройства. Люди в сельском районе Пьютхан (208 из 268) чаще имели доступ к медицинскому обслуживанию, чем жители района Читван (133 из 241).

**Вывод:**

Внедрение этого инструмента привело к более широкому использованию служб охраны психического здоровья в странах с низким уровнем доходов, располагающих ограниченными ресурсами здравоохранения. Инструмент продемонстрировал эффективность в сельских районах, где общины сплоченные, и общественные информанты знакомы с теми, кто нуждается в услугах службы охраны психического здоровья.

## Introduction

Globally, underutilization of mental health services is a major barrier to reducing the burden of disease attributable to mental, neurological and substance-use disorders.^1^ Service underutilization has been attributable to lack of awareness of service availability; lack of recognition of mental, neurological and substance-use disorders in oneself or one’s family; stigma against seeking mental health care; and perceived ineffectiveness of treatments.^2^ Routine or indicated primary health-care screening has been proposed to tackle this challenge, but this approach misses people who rarely use primary health-care services. In areas with high poverty levels and/or long travel times to health facilities, large portions of the population access primary care infrequently. Moreover, many low- and middle-income countries lack resources for widespread screening, especially in populations with high illiteracy that require health staff to administer screening tools.

An alternative approach to increase utilization is community case detection, which employs a gate-keeper model where people with regular community engagement are taught to identify and refer people for assessment and treatment in primary health care. However, community case detection has received limited attention for mental health.

To address this challenge, we developed a community informant detection tool, which we piloted in Nepal.^3^ The tool facilitates detection of people with depression, alcohol-use disorder, epilepsy and psychosis and helps identified people to seek care. The disorders were selected based on prevalence, burden of disease and responsiveness to evidence-based treatments, and have been confirmed for Nepal through an expert priority-setting study.^4^ The tool is developed on the premise that people who are intimately connected within the community, such as community health workers (CHWs), are in a position to identify those in need of care, if they are provided with a tool for identification. The structured tool contains vignettes, which are sensitive to the context, rather than symptom checklists and uses pictures that are easy to understand for low literacy populations. Trained lay community informants (e.g. CHWs or civil society women’s groups), use the tool during daily routine activities, where they check the extent to which people match paragraph-long vignettes using a four-point scale. The pictorial vignettes are designed to initiate help-seeking for mental health treatment in primary care settings. The community informants do the vignette matching based on their observation of people as part of their interactions during their regular responsibilities. If the person fits well with the description, they will ask additional questions on need for support or impairment in functioning. The questions are an integral part of the tool with yes/no responses, functioning as a decision flowchart. In the case of a positive reply to either of the two questions, the informant encourages the person (possibly through their family) to seek help in health-care facilities where mental health services are being offered and the person can be evaluated by trained health professionals. No stigmatizing psychiatric labels are used and encouragement for help-seeking is targeted to observable behaviours and signs of distress.

Previous studies demonstrated that the tool has an accuracy comparable to primary health-care screening in high-income countries and better than standard screening tools in Nepal (positive predictive value of 0.64 and negative predictive value of 0.93).^3^^,^^5^

Here we determine whether application of the tool increases help-seeking behaviour among people who would otherwise be unlikely to seek care. We also assessed how many of the referred people pursued primary health-care services and started on treatment.

## Local setting

Decentralized mental health services are scarce in Nepal^4^ and less than 5% of people with alcohol-use disorder and less than 10% with depression seek treatment (Luitel et al., Transcultural Psychosocial Organization Nepal, unpublished data, 15 March 2017).

The study took place in two Nepalese districts. Chitwan district in southern Nepal has been the implementing site for the Programme for Improving Mental Healthcare (PRIME) since 2011.^6^^,^^7^ The district is densely populated, is relatively well resourced and at the time of the study had 12 health-care facilities with mental health services. Pyuthan is a more remote and poorer hill district, and was the site for the Mental Health Beyond Facilities (mhBeF) initiative from 2013 to 2015. When the study was conducted, the district had six facilities providing mental health services.

Both mental health programmes were implemented by the nongovernmental Transcultural Psychosocial Organization (TPO) Nepal.

## Approach

In 2014, community informants residing in the study areas were selected based on their interest in participating. In addition, the district public health office recommended CHWs. The informants received two-days of training in TPO’s offices in both districts. The training consisted of how to use the tool and ethical issues associated with case-finding, confidentiality and how to encourage, but never impose help-seeking. In their routine work, the informants then used the tool to identify people with mental disorders proactively. For the purpose of the study, people identified were given a referral slip to visit a health facility with staff trained in mental health services (following the mhGAP intervention guide).^8^ The informants filled in the contact information for the identified person and themselves on referral slips, while information on the location of the appropriate health facility was provided verbally. The visits were free of cost.

All 674 people, identified with the tool between April and May 2014, were scheduled to be visited by a research assistant three weeks after the date of detection. The people, who the informants were able to reach after three weeks and who provided consent to participate in the study, were asked whether they had visited a health-care facility in the past three weeks. The participants who answered “yes” were also asked the following questions: Who or what determined whether you sought help (including referral through the tool as one of the options)? What problem did you seek help for? Was treatment initiated, if so what treatment? Additionally, we asked participants for sociodemographic characteristics. For the participants that accessed health care, we cross-checked their answers with their clinical diagnosis and treatment records and we checked the clinical records for completeness.

We obtained ethical approval for this study from the Nepal Health Research Council.

## Relevant changes

**Fig. 1:**
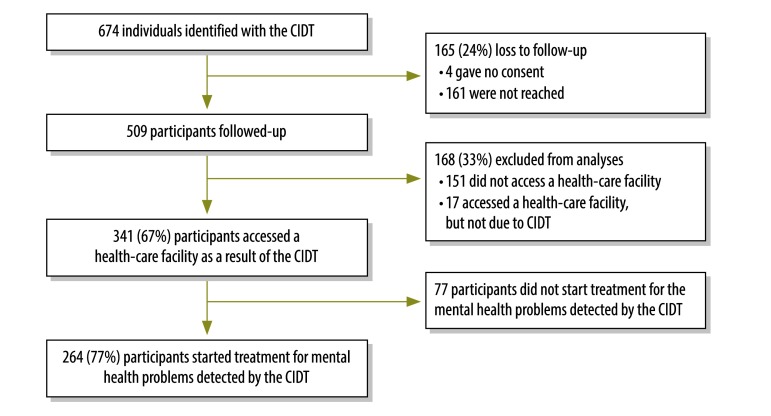
Flowchart for the proactive community case-finding of individuals with suspected mental disorders, Nepal, 2014

Out of the 509 participants, 67% (341) accessed a health-care facility after being referred as a result of the proactive detection approach. We excluded 17 participants that accessed health care, but who did not explicitly mention this was because of the tool (Fig. 1). Among the 341 participants accessing care, 264 (77.0%) received diagnoses and started treatment for mental illness: 34.8% (92) received diagnoses for epilepsy; 31.1% (82) for psychoses; 15.9% (42) for depression; 14.0% (37) for alcohol-use disorder; 2.3% (6) for anxiety; and 1.9% (5) for being bipolar (total exceeds 100% because of comorbidity). In Chitwan, 55.2% of participants (133 out of 241) accessed care, while the percentage in Pyuthan was 77.6 (208 out 268). The mental health services offered by trained primary health-care workers included pharmacological and psychosocial interventions.^7^ Participants were mainly referred by female community health volunteers (84.9%) and civil society women’s groups (14.5%).

CIDT: community informant detection tool.

Those who accessed health care versus those who did not had similar age, gender, education and marital status (Table 1). The people that accessed care had significantly longer distance to the nearest health-care facility than those not accessing care (*P* < 0.001; *t*-test).

**Table 1:** Sociodemographic characteristics of individuals detected with community informant detection tool for mental disorders, Nepal, 2014

Characteristic	Accessed health care (*n* = 341)	Did not access health care (*n* = 168)	Pyuthan district (*n* = 268)	Chitwan district (*n* = 241)	Total (*n* = 509)
**Age, mean years (SD)**	35.9 (16.8)	40.1 (16.5)	32.9 (17.4)	42.1 (14.7)	37.3 (16.8)
**Female, no. (%)**	174 (51.0)	77 (45.8)	154 (57.5)	97 (40.2)	251 (49.3)
**Mins walking to clinic, mean (SD)**	56.8 (51.7)	38.5 (39.2)	76.1 (54.4)	22.5 (14.0)	50.7 (48.6)
**Education, no. (%)**					
No formal school	147 (43.1)	70 (41.7)	112 (41.8)	105 (43.6)	217 (42.6)
Primary school	97 (28.4)	52 (31.0)	77 (28.7)	72 (29.9)	149 (29.3)
Secondary school	89 (26.1)	35 (20.8)	75 (28.0)	49 (20.3)	124 (24.4)
College	8 (2.3)	11 (6.5)	4 (1.5)	15 (6.2)	19 (3.7)
**Marital status, no. (%)**					
Unmarried	101 (29.6)	41 (24.4)	88 (32.8)	54 (22.4)	142 (27.9)
Married	211 (61.9)	113 (67.3)	158 (59.0)	166 (68.9)	324 (63.7)
Widow	20 (5.9)	10 (6.0)	14 (5.2)	16 (6.6)	30 (5.9)
Divorced/separated	9 (2.7)	4 (2.4)	8 (3.0)	5 (2.1)	13 (2.6)
**Type of informant, no. (%)**					
FCHV	283 (83.0)	149 (88.7)	191 (71.3)	241 (100.0)	432 (84.9)
Women’s group	56 (16.4)	18 (10.7)	74 (27.6)	0 (0.0)	74 (14.5)
Traditional healer	2 (0.6)	0 (0.0)	2 (0.7)	0 (0.0)	2 (0.4)
Youth group	0 (0.0)	1 (0.6)	1 (0.4)	0 (0.0)	1 (0.2)
**Identified vignette, no. (%)^a^**					
Depression	92 (27.0)	55 (32.7)	88 (32.8)	59 (24.5)	147 (28.9)
Psychosis	90 (26.4)	31 (18.5)	55 (20.5)	66 (27.4)	121 (23.8)
Epilepsy	119 (34.9)	43 (25.6)	125 (46.6)	37 (15.4)	162 (31.8)
Alcohol-use disorder	40 (11.7)	39 (23.2)	0 (0.0)	79 (32.8)	79 (15.5)

FCHV: female community health volunteer; SD: standard deviation.

^a^ This indicates the condition identified by the community informant and recorded on the community informant detection tool; it does not indicate the clinical diagnoses made by health workers at health-care facilities.

Note: Inconsistencies arise in some values due to rounding.

## Discussion

Our results combined with data from our previous study^3^ demonstrate a two-thirds by two-thirds effect of identifying and treating people with mental disorders, when using the tool. First, community informants accurately detected in two-thirds of the cases.^3^ Second, two-thirds of those detected initiated help-seeking and went on to access health care. These results indicate that the access gap for mental health care can be reduced by intervening on the demand-side, suggesting that this tool could be useful in other low- and middle-income countries experiencing low-treatment coverage for mental illness (Box 1).^9^

Box 1 **Summary of main lessons learnt**

- Using a community informant detection tool decreases the access gap to treatment for mental disorders.
- The tool seems beneficial in rural settings, where communities are close-knit and community informants are familiar with those in need of mental health services.
- The task of case identification can then be shifted to community members outside the health-care system as a way to broaden access to mental health care.

Participants from Pyuthan were more likely to access health care than those living in Chitwan, even though they had longer distances to travel to health facilities. This result suggests that the tool is especially beneficial in more rural settings, where communities are close-knit and CHWs and other liaisons are familiar with those in need of mental health services. In rural communities, residents may be more likely to trust and follow the recommendations of CHWs. In addition, Pyuthan had no local access to treatment for mental disorders before the implementation of the mhBeF programme, so the tool may perform better when health services are newly initiated.

This study has limitations, of the people initially identified as potentially having a mental disorder according to the tool, 24% were excluded, mostly because they could not be reached after three attempts by research staff. Also, this study depended on participants’ recall of what triggered them to seek care.

This study demonstrates that the structured and context-sensitive detection tool supports community informants in proactive case-finding. The informants’ tacit knowledge and awareness of who is suffering in the community helps them to identify people in need of mental health care. This approach is useful in places where mental health care is newly established and heavily stigmatized due to lack of awareness; it also helps to support disadvantaged groups who face more barriers due to social and economic vulnerabilities.^10^^,^^11^

The Nepalese government has included the tool in national health care packages ^12^ and the approach has been scaled-up to other districts during the emergency response following the 2015 earthquakes.

To increase coverage of mental health care in low- and middle-income countries, efforts to overcome supply-side and demand-side barriers should occur simultaneously; this includes shifting tasks from mental health professionals to CHWs.^13^ Whereas the supply-side requires increased service delivery models, the demand-side requires stimulatingdemand, for example through proactive community case-finding. Inclusion of the community informant detection tool or similar case detection interventions in service delivery models, could address the treatment and access gaps for mental health in low- and middle-income countries.
